# Vulvar High-Grade Squamous Intraepithelial Lesions Treated with Imiquimod: Can Persistence of Human Papillomavirus Predict Recurrence?

**DOI:** 10.3390/cancers14194808

**Published:** 2022-09-30

**Authors:** Maria-Eulalia Fernández-Montolí, Fatima Heydari, Fabrizia Lavecchia, Miquel-Ângel Pavón, Esther Guerra, Xavier Matias-Guiu, Maria-Dolores Marti, Sara Tous

**Affiliations:** 1 Gynecology Department, Hospital Universitari de Bellvitge, IDIBELL, Universitat de Barcelona, L’Hospitalet de Llobregat, 08907 Barcelona, Spain; 2 Medicine and Translational Research Doctorate Program, Faculty of Medicine and Health Sciences, University of Barcelona, 08036 Barcelona, Spain; 3 Infections and Cancer Laboratory, Cancer Epidemiology Research Program, Catalan Institute of Oncology (ICO), Bellvitge Biomedical Research Institute (IDIBELL), L’Hospitalet de Llobregat, 08908 Barcelona, Spain; 4 Centro de Investigación Biomédica en Red de Epidemiología y Salud Pública (CIBERESP), 28029 Madrid, Spain; 5 Pathology Department, Hospital Universitari de Bellvitge, IDIBELL, L’Hospitalet de Llobregat, 08907 Barcelona, Spain; 6 Cancer Epidemiology Research Program, Catalan Institute of Oncology (ICO), Bellvitge Biomedical Research Institute (IDIBELL), L’Hospitalet de Llobregat, 08908 Barcelona, Spain

**Keywords:** vulvar high-grade squamous intraepithelial lesion, vulvar intraepithelial neoplasia, human papillomavirus, imiquimod, recurrence, vulvar carcinoma, immunity

## Abstract

**Simple Summary:**

Vulvar high-grade intraepithelial lesion (vulvar HSIL) is a premalignant vulvar condition that requires intervention, usually surgery. It recurs frequently, and its treatment involves repeated disfiguring surgeries. Vulvar HSIL is associated with human papillomavirus. Imiquimod is a medical treatment option currently attracting attention because vulvar high-grade intraepithelial neoplasia is frequent in young women with multiple vulvar lesions. Few studies have evaluated the long-term effects of the response to imiquimod and the association of human papillomavirus with response and recurrence. We describe a retrospective (with cases already treated) study designed to determine the long-term response to imiquimod in patients with vulvar HSIL, and also to analyze the role of human papillomavirus (HPV), and different HPV types, in the persistence or recurrence of vulvar HSIL after imiquimod treatment.

**Abstract:**

**Objectives:** Vulvar high-grade squamous intraepithelial lesion (vulvar HSIL) or vulvar intraepithelial neoplasia (VIN) is a premalignant condition that can progress to carcinoma. Imiquimod is a topical drug with high effectiveness and low morbidity. We aimed (1) to assess the long-term response to imiquimod in a cohort of patients with vulvar HSIL and (2) and to analyze the role of HPV determined in pre- and post-imiquimod treatment biopsies in the persistence or recurrence of vulvar HSIL. **Design:** Retrospective study between 2011 and 2022. **Setting**: Referrals from the primary care area of Baix Llobregat treated in the gynecology department of a university hospital in Barcelona, Spain. **Population**: 20 women with vulvar HSIL treated with imiquimod. **Methods:** The inclusion criteria were vulvar HSIL, vulvar HPV determination by pre- and post-treatment biopsy, acceptance of medical treatment, at least one follow-up and 4 weeks of treatment. **Main outcome measures**: Histological diagnosis of vulvar HSIL with pre- and post-imiquimod HPV determination. Response to treatment (complete, partial, no response, recurrence). **Results:** After imiquimod, 10 (50%) and 6 (30%) cases had complete and partial responses, respectively. Another 4 cases (20%) did not respond. Before treatment, 19 (95%) cases were positive for vulvar HPV (16 cases had HPV type 16). After treatment, 10 cases (50%) were positive for HPV (8 cases with HPV type 16): 2 cases (20%) with a complete response, 5 cases (83.3%) with a partial response and 3 cases (75%) with no response. Eight of the 10 HPV-negative cases (80%) post-treatment showed a complete response. HPV type 16 was present in 16 cases (84.2%) pre-treatment and in 8 cases (80%) post-treatment. Ten patients underwent additional treatments following a partial response, no response or recurrence. The 2 HIV and 3 immunosuppressed patients treated with imiquimod showed a partial response and required additional treatment. All these patients were HPV-positive pre- and post-treatment (100%). Response to imiquimod was associated with post-treatment vulvar HPV positivity (*p* = 0.03). The median time to a complete response in HPV-negative cases was 4.7 months versus 11.5 months in HPV-positive cases post-imiquimod treatment. Recurrence of vulvar HSIL was observed in 7 patients (35%), with a median time to recurrence of 19.7 months (range 3.2–32.7). Recurrence was experienced in 10% of cases with a complete response, in 4/6 (66.6%) cases with a partial response, and in 2/4 (50%) women with no response. Four of the 7 recurrent cases (57%) were infected with HIV or immunosuppressed. Six (85%) of the recurrent cases were HPV-positive post-treatment (all were HPV type 16). Four (30.7%) of the non-recurrent cases were HPV-positive post-treatment with imiquimod (*p* = 0.05), two of which were HPV type 16 (50%). **Conclusions:** Imiquimod effectively treats vulvar HSIL. Cases with a complete response showed less HPV positivity post-treatment than partial or non-response cases. Recurrences were more frequent in those with a partial or no response to imiquimod, and in immunosuppressed patients. In recurrent cases, 85% were HPV-positive post-treatment, while 30.7% of non-recurrent cases were HPV-positive. HPV positivity in the post-treatment biopsy suggests the need for stricter follow-up of patients.

## 1. Introduction

Vulvar high-grade squamous intraepithelial lesion (vulvar HSIL), or vulvar intraepithelial neoplasia (VIN), is a premalignant condition that can progress to carcinoma [1]. Two forms of squamous intraepithelial lesion exist depending on their etiopathogenetic pathway: vulvar HSIL and differentiated VIN. Vulvar HSIL refers to lesions strongly associated with human papillomavirus infection (HPV) [2,3]. Differentiated VIN (dVIN) refers to lesions that are not associated with HPV infection [3] and is related to chronic inflammatory dermatoses, such as lichen sclerosus and lichen simplex chronicus [4].

Treatment is recommended for vulvar HSIL because it is a premalignant condition [5]. However, the classic surgical treatment produces high morbidity and recurrence rates [6]. This previously required extensive and repeated disfiguring surgeries to eradicate the lesions, while current treatment strategies aim to prevent progression to vulvar carcinoma, preserve normal anatomy, relieve symptoms, and maintain both quality of life and sexual function [7]. Medical treatment has therefore arisen as a treatment option for vulvar HSIL, particularly because it usually presents as multifocal lesions in young women [4].

Imiquimod, a topically administered immune response modifier drug that has proven effectiveness and low morbidity, is considered a first-line therapy for vulvar HSIL [4]. It is a Toll-like receptor 7 agonist that activates immune cells and stimulates the concentration of pro-inflammatory cytokines [8]. Reported results for the response of vulvar HSIL to imiquimod ranged from 5% to 88% in 13 of 14 studies in a review that included 780 women [9].

Given that vulvar HSIL development strongly correlates with HPV infection, clearing the infection with imiquimod therapy should improve the pathological response. Few studies have analyzed HPV clearance by imiquimod. A systematic review found that HPV clearance ranged from 33% to 85% [9], while a study by van Seters showed that 12 of 14 cases in which imiquimod cleared HPV showed a complete response of VIN [10]. Furthermore, HPV clearance at 4 weeks after imiquimod normalized immune cell counts and p16 INK 4a [11]. Although HPV clearance in the vulva could lead to fewer recurrences, Westermann observed that recurrence was more frequent after imiquimod treatment in HPV-positive cases (38%) than in HPV-negative cases (11%) [12]. Few studies have also evaluated the long-term effects of the response to imiquimod; one study [13] followed 24 patients included in the trial by van Seters [10] for a median of 7.2 years. The duration of follow-up is essential when comparing the reported recurrence rates: 6.8% at 6 months and up to 50% (14th year of follow up) [7]. Research has also largely ignored the HPV genotypes in biopsy samples before and after imiquimod treatment or the association with pathologic response and recurrence in long-term follow-up, and has included only vulvar HSIL cases. Two RCTs comparing imiquimod with placebo performed a post-treatment biopsy and HPV assessment at 20 weeks [10] or only biopsy [14].

We aimed to assess the long-term response to imiquimod in a cohort of patients with vulvar HSIL treated in our hospital, and to analyze the role of HPV determined in pre- and post-imiquimod treatment biopsies in the persistence or recurrence of vulvar HSIL.

## 2. Materials and Methods

### 2.1. Study Design

This was a retrospective, single-center cohort study of consecutive adult patients diagnosed with vulvar HSIL by vulvar biopsy and referred to the Gynecology Department, University Hospital of Bellvitge (Barcelona, Spain) from the primary care area of Baix Llobregat (ASSIR) between March 2011 and 2022. We included patients with a histological diagnosis of vulvar HSIL (HPV-associated squamous intraepithelial lesions), treated with imiquimod for at least 4 weeks and with at least 1 follow-up visit. We excluded the histological diagnosis of differentiated VIN, vulvar Paget’s disease, vulvar carcinoma; patients with vulvar LSIL (VIN 1) or a negative (vulvar epithelium without pathology) post-treatment biopsy result; and patients who did not complete at least 4 weeks of treatment or who had no follow-up; refusal of patients to participate, or patients without post-treatment biopsy.

### 2.2. Vulvar Biopsy

A 4 mm punch biopsy was performed before treatment. When necessary, women underwent more than one biopsy pre- or post-treatment to exclude invasive carcinoma. The histologic analyses of the vulvar biopsy specimens were performed according to the World Health Organization (WHO) [15]. When the pre-treatment biopsy was performed in another hospital, a gynecological pathologist reviewed the biopsy specimen in our hospital. After completion of treatment, biopsies were taken in all patients. Treatment was terminated if a clinically complete response was observed at vulvoscopy or after 32 weeks of treatment in the case of a partial or no response. If there was a complete response, biopsy was performed at the site of the previous lesion (according to photographs and medical records).

Two gynecological pathologists analyzed all the biopsies.

### 2.3. Imiquimod Treatment

Imiquimod 5% cream was administered topically as a thin layer of the cream left on for 6–8 h at night and then washed off with soap and water. Treatment was prescribed 3 days a week for 16 weeks or until lesion disappearance or for up to 32 weeks. If severe or moderate side effects developed, treatment was stopped temporarily and resumed after 1–2 weeks. Patients could also reduce the frequency of administration to 1 or 2 days a week.

### 2.4. Follow-Up

We scheduled follow-up at 4 weeks after the start of treatment and every 2 months thereafter. At each visit, we evaluated treatment efficacy, side effects, and symptoms, and we confirmed adherence to treatment. This included physical examination and vulvoscopy with a description of the size, color, number, and location of any lesions. Vulvar intraepithelial lesions were measured with a ruler and photographs were taken at the first visit and after treatment. Post-treatment biopsies were obtained by a 4 mm dermatological punch at the end of treatment, when lesions increased in size, or ulcers were present. If lesions reappeared during follow-up, post-treatment biopsy was repeated. After the prescribed treatment (medical or surgical), follow-up was conducted by periodic visits every 6 months. Surgical treatment with wide excision was performed in all cases with a partial response or lack of response to imiquimod.

### 2.5. Clinical Response

Clinical response to treatment was defined as a reduction in the lesion size after treatment with imiquimod. Complete response was defined as a 100% reduction in the lesion size with negative post-treatment biopsy for vulvar HSIL, partial response as a >25% reduction in lesion size, and no response as a ≤25% to 0% reduction [10]. Recurrence was defined as a histological diagnosis of vulvar HSIL during follow-up after either a previous complete response or surgical excision.

### 2.6. HPV Determination

Four paraffin sections were obtained for each formalin-fixed paraffin-embedded (FFPE) block. The first and last sections (3-mm thick) were used for hematoxylin and eosin staining, and the second and third intervening sections (5-mm thick) were used for HPV testing and genotyping (sandwich method) [16]. DNA was extracted from FFPE sections using the Maxwell 16 FFPE Plus LEV DNA Purification kit (Promega Corp., Madison, WI, USA) and eluted with 100 μL of nuclease-free water. HPV detection and genotyping was performed using 10 μL of sample DNA and the Anyplex™ II HPV28 assay (Seegene, Seoul, South Korea). This assay detects 28 HPV genotypes, including HR-HPV (HPV-16, -18, -31, -33, -35, -39, -45, -51, -52, -56, -58, -59 and -68), LR-HPV (HPV-6, -11, -40, -42, -43, -44, -53, -54, and -70), and possible carcinogenic genotypes (HPV-26, -61, -66, -69, -73, and -82). We automated the data recording and interpretation with the Seegene viewer software (Seegene, Seoul, South Korea) and processed FFPE blocks under strict physical separation pre/post polymerase chain reaction, while systematically testing blank paraffin blocks in parallel to monitor for contamination [17].

### 2.7. Outcomes

For the primary outcome, we report the clinical and pathological response to imiquimod, the recurrence of vulvar HSIL, based on HPV determination in vulvar biopsies before and after imiquimod treatment. For the secondary outcomes, we report the presence of side effects, requirement for treatment-free periods, smoking, age, immunosuppression, lesion size and type, and previous vulvar HSIL lesions and treatment. Finally, we recorded and classified side effects as mild, moderate and severe.

### 2.8. Data Analysis

An electronic case report form was designed in Microsoft Access and data from the consecutive patients were entered. Information on follow-up was retrieved at the end of the study period. 

The database was verified to evaluate the quality of the data collected in the study. We used IBM SPSS (version 25.0) (Armonk, NY, USA) for data processing and analysis. 

For the descriptive analysis, categorical variables are described as the number of cases and percentage, whereas continuous variables are described as the median (min and max), and the first and third quartiles, as appropriate. Because of the small sample size, we applied non-parametric tests and calculated significant differences as two-sided *p*-values of <0.05. We used chi-square or Fisher exact tests to analyze differences in categorical variables between groups and the Kruskal–Wallis test for differences in continuous variables.

### 2.9. Ethical Aspects

This manuscript was reviewed by the Research Ethics Committee of the University Hospital of Bellvitge. The requirement for informed consent was waived, given that this observational study consists of a retrospective analysis of our usual healthcare practice. Patients’ clinical data were pseudonymized in order to maintain confidentiality in accordance with the provisions of Regulation (EU) 2016/679 of the European Parliament and of the Council of 27 April 2016 on the protection of natural persons with regard to the processing of personal data and on the free movement of such data, and repealing Directive 95/46/EC (General Data Protection Regulation) and the Organic Law 3/2018, of December 5, Protection of Personal Data and Guarantee of Digital Rights. This study respects the principles of the Declaration of Helsinki (Fortaleza, 2013). The study was approved by the Clinical Research Ethics Committee of Bellvitge University Hospital (Reference EOM025/22).

## 3. Results

### 3.1. Study Cohort

**Figure 1:**
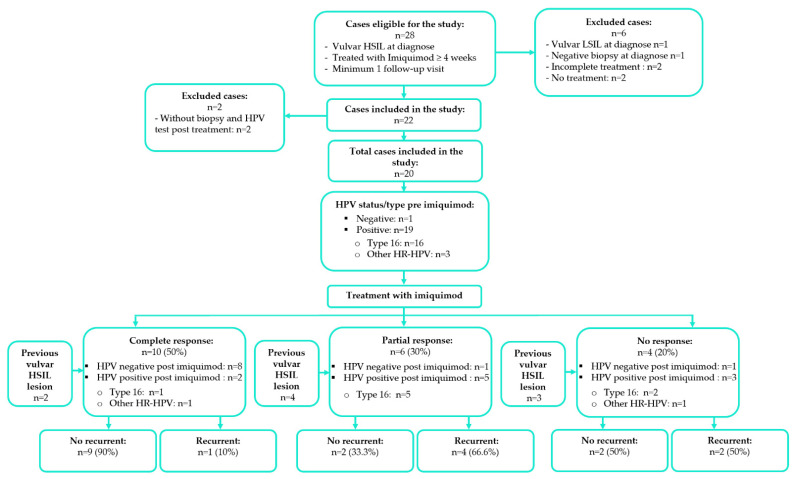
Study inclusion and exclusion of patients treated with imiquimod for vulvar high-grade squamous intraepithelial lesion (vulvar HSIL). Different types of response after imiquimod treatment, HPV status and type determined pre- and post-imiquimod treatment, history of previous vulvar HSIL lesions, and recurrent vulvar HSIL after imiquimod treatment.

Figure 1 shows the flow chart for study participation. We enrolled 28 consecutive adult women with vulvar HSIL. Of these, we excluded 1 with vulvar LSIL (VIN 1), 1 misclassified with vulvar HSIL (negative pre-treatment biopsy), 2 who received incomplete treatment, and 2 who refused medical treatment. Another 2 cases had no post-treatment biopsy. Thus, 20 of the 28 eligible cases were included (71.4%). Table 1 provides a summary of the patient characteristics (median age, 50 years; range, 27–87 years). The median follow-up time was 37.3 months (range 1–89.1), with 75% of patients followed for over 64.2 months. The median time interval between the pre- and post-imiquimod biopsy was 7.3 months.

**Figure 2:**
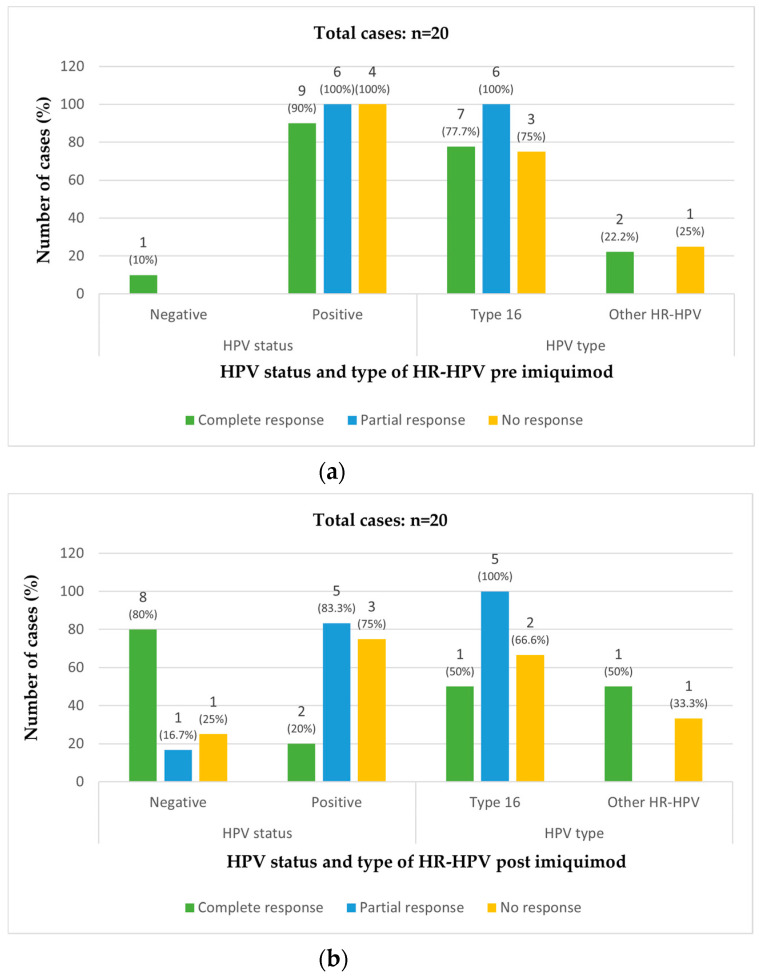
Number and percentage of cases of HPV and type of HR-HPV in the biopsies pre- and post-treatment with imiquimod in patients with complete, partial, and no response to treatment: (**a**) In HPV status, numbers in histogram and percentages are shown separately for each response. E.g., in complete response there are 10 cases where 1 (or 10%) and 9 (or 90%) correspond to negative and positive cases, respectively. In HPV type, numbers in histogram and percentages are obtained from positive HR-HPV cases inside each response separately (i.e., in complete response, there are 7 cases of type 16 or 77.7%, and 2 cases of other HR-HPV or 22.2% among the 9 positive HR-HPV cases). (**b**) In HPV status, numbers in histogram and percentages are shown separately for each response. In HPV type, numbers in histogram and percentages are obtained from positive HR-HPV cases inside each response separately. Ten cases received additional treatment following imiquimod treatment. Complete response: *n* = 10, Partial response: *n* = 6, No response: *n* = 4.

Vulvar HPV was positive in the pre-treatment biopsy in 19/20 cases (95%). The only case that was HPV-negative pre-treatment showed a complete response. HPV type 16 was present in 16 (80%) pre-treatment vulvar biopsies. Another 3 cases (15%) had either type 18, 53, or 51 HPV (Table 2) (Figure 2a).

After treatment, 10 cases (50%) were vulvar HPV-positive (8 cases with HPV type 16). Two cases (20%) among those with a complete response, 5 cases (83.3%) among those with a partial response and 3 cases (75%) among those showing no response were HPV-positive post-treatment. Eight of the 10 cases that were HPV-negative post-treatment (80%) showed a complete response. HPV type 16 was found in 16 cases (84.2%) pre-treatment, and in 8 cases (80%) post-treatment (Table 2) (Figure 2b).

### 3.2. Treatment Success and Factors Associated with Response to Imiquimod

Among the 20 included cases, 10 (50%) showed a complete pathological and clinical response and 6 (30%) showed a partial response, totaling 16 cases (80%) with a response. Among the 4 cases (20%) classed as not responding, 1 case showed a reduction in lesion size of <25% and 1 case progressed to vulvar carcinoma.

The median total duration of imiquimod treatment was 14.5 weeks (range 4–32 weeks), and the median time to clinical response was 4.9 months (range 1–37.5 months). 

The median follow-up time in complete response cases was 37.3 months and the median time to response (post-imiquimod biopsy) was 4.7 months (Table 3).

Our cohort included vulvar HSIL with a median pre-treatment lesion size of 20 mm (range 5–60 mm), classified as unifocal in 5 cases (25%), isolated multifocal in 6 cases (30%), and multifocal in 9 cases (45%). Nine patients (45%) had previous vulvar HSIL lesions (Table 2). After treatment with imiquimod, the lesions showed a median reduction in size of 93.7% (range 0–100%).

Among the 10 patients with a complete response, 9 had a negative post-treatment biopsy (45%) and 1 had vulvar LSIL (5%), while 8 were HPV-negative post-treatment (80%) and 2 were HPV-positive (20%) (1 type 16 and 1 type 56) (Table 2) (Figure 2a,b).

In those with a complete response, the median time to biopsy (response) in patients that were HPV-negative post-treatment was 4.7 months (range 1–16.5), while in HPV-positive patients it was 11.5 months (range 2.2–20.9) (2 cases).

Vulvar HSIL persisted after treatment in 9 cases (45%), with a partial response in 6 cases (30%) and no response in 4 cases (20%). All cases with a partial or no response were HPV-positive in the pre-treatment biopsy. Pre-treatment, HPV 16 was found in all 6 cases (100%) that subsequently had a partial response to treatment, and in 3 out of the 4 cases (75%) with no response to imiquimod treatment (Table 2) (Figure 2a).

After treatment, HPV was positive in 5 out 6 cases (83.3%) with a partial response and in 3 out of 4 cases (75%) with no response. Post-treatment, HPV 16 was found in 5/6 (83.3%) cases that showed a partial response and in 2/4 (50%) cases that showed no response (Table 2) (Figure 2b). Vulvar HSIL progressed to squamous carcinoma of the vulva in 1 non-response case (5%).

Imiquimod produced frequent side effects (55%), causing 75% of patients to stop the treatment temporarily (10 cases; 50%) or permanently (5 patients; 25%). Two women reported burning, pain, and stinging (10%), but all symptoms were mild or moderate, and 9 women (45%) had no symptoms. After applying imiquimod, we observed symptoms in the vulvar skin of 12 cases (60%), erythema being the most frequent (6 cases; 30%) (Table 4).

Regarding immune status, 2 patients were HIV positive, and both had a partial response. One was lost to follow-up and progressed to cancer, while the other underwent a surgical procedure for recurrence before later receiving cidofovir. Both patients had HPV type 16 before and after imiquimod therapy. Three women had immunosuppression (kidney transplant, combined immunodeficiency, lymphomatoid papulosis, treated with methotrexate, and micro-leukemia). All had a partial response and underwent surgery after imiquimod but showed persistence necessitating further surgeries. All patients presented vulvar HPV 16 positivity before and after imiquimod (1 case had type 16 and 56 at both assessments).

Data on immunosuppression, vulvar HPV positivity after imiquimod, vulvar HPV type and previous vulvar HPV surgery are shown in Table 1 and Table 2, Figure 2a,b.

In our series, 10 patients underwent additional treatments during follow-up. In complete response cases, only 1 case recurred and had additional treatments. Neither of the 2 cases that were HPV-positive post-treatment had additional treatments (Table 5).

### 3.3. Partial Response of Vulvar HSIL to Imiquimod

Six patients showed a partial response after treatment with imiquimod. The post- treatment median size of the lesions was 8.5 mm (range 3–20 mm). Two cases had a unifocal lesion, 3 had multifocal lesions, and 1 had isolated multifocal lesions. In these cases, the size of the lesions decreased by 57% (median, 53.3%; min–max, 33.3–87.5%). 

The median follow-up time was 38.2 months, the median time of post-treatment biopsy (time to response) was 5.8 months, and the median time to recurrence was 16.2 months (Table 3).

Five cases underwent surgical excision. The post-excision pathological diagnoses were vulvar HSIL (4 cases) and vulvar LSIL (1 case), with the remaining case (HSIL) lost to follow-up and later found to have progressed to vulvar carcinoma. 

The 5 cases with vulvar HSIL were positive for HPV type 16 (1 case was also positive for type 56) after imiquimod treatment. The HPV-negative case corresponded to the case with vulvar LSIL (Table 2) (Figure 2b).

The case with vulvar LSIL had free surgical excision margins and did not recur. However, of the 4 cases with vulvar HSIL, 2 had involved margins, 1 had uncertain margins, and another had missing margins in the pathology report. All 4 cases persisted or recurred, necessitating further surgical procedures, though 1 was treated with cidofovir (Table 2). The woman with vulvar carcinoma who was lost to follow-up had a history of poor attendance, HIV infection, and drug dependence. After 23.3 months, she presented to another hospital with a lesion that had progressed to stage IV vulvar cancer.

Regarding additional treatments, all 4 cases with a partial response recurred, and all had additional treatments after imiquimod. All 5 cases that were HPV positive post-treatment received additional treatments (Table 5 and Table 6).

### 3.4. No Response of Vulvar HSIL to Imiquimod

Four cases had little or no response to imiquimod (including 1 case with a response of <25%), among which one progressed to carcinoma of the vulva. One case had a unifocal lesion, 1 had 2 isolated multifocal lesions, and 2 had multifocal lesions. The median lesion size post-treatment was 10 mm (min–max 10–80), with a 23.1% reduction achieved in one case and the remaining cases showing no reduction.

The median follow-up time was 42 months, the median biopsy time was 3.3 months, and the median time of recurrence of vulvar HSIL was 22.1 months (Table 3).

Three of these cases underwent surgical excision and the pathological report indicated vulvar HSIL in 2 cases and carcinoma in 1 case. 

Vulvar HPV was found in 3 cases (75%) post-imiquimod treatment and was type 16 in the 2 cases of HSIL and type 52 in the vulvar carcinoma (Table 2) (Figure 2b). Surgical excision margins were involved in both HSIL cases, but the carcinoma specimen showed free margins. Recurrences were observed in the 2 HSIL cases treated with surgery.

The case that was not treated was negative for vulvar HPV post-treatment (Figure 2b). This case had dementia and was lost to follow-up. The general practitioner’s records indicated that the patient was still alive and was not complaining of vulvar problems.

Regarding the additional treatments post-imiquimod, all 3 cases with no response recurred and all had additional treatments. Similarly, all 3 cases that were HPV-positive post-treatment had additional treatments (Table 5 and Table 6).

### 3.5. Recurrence of Vulvar HSIL and Factors Associated with Recurrence

Recurrence occurred in 7 patients (35%), with a median time to recurrence of 19.7 months (range 3.2–32.7) (Table 3). The recurrence rate observed by response type approached significance (*p* = 0.06) (Table 2). Four of these cases (57%) had HIV infection or immunosuppression.

Among the 10 cases with a complete response, only 1 case (10%) had recurrence (negative for vulvar HPV after imiquimod) (Table 7). The patient had surgical excision with a negative pathological result and free margins. She received a second treatment with.

Imiquimod, after which she had a negative biopsy post-treatment. By contrast, 4 of the 6 cases with a partial response had recurrence (66.6%), while 2 of the 4 patients with no response (one with response <25%) had recurrence (50%) (Table 2).

The factors associated with recurrence of vulvar HSIL were immunosuppression (*p* = 0.03), previous vulvar HSIL lesions (*p* = 0.017), previous surgical treatment for vulvar HSIL (*p* = 0.02), positive HPV after imiquimod (*p* = 0.057), and HPV type after imiquimod (*p* = 0.01) (Table 8) Age, smoking habit, HIV status, HPV status and HPV type pre-imiquimod, initial type of lesion, initial size of lesion, type of lesion post-imiquimod, size of lesion post-imiquimod, side effects (signs, symptoms), reduction of dose and cessation of treatment had no significant effect on outcome.

Among the recurrent cases, HPV was found in 7 cases (100%) pre-treatment, being HPV type 16 in all cases. After imiquimod treatment, HPV was positive in the biopsy in 6 out of 7 cases (85.7%), all of which were HPV 16-positive (100%) (Table 7). 

Among the non-recurrent cases, 12/13 cases (92.3%) were HPV-positive pre-treatment; HPV type 16 was found in 9 of these cases (75%). Three cases were positive for other HPV types (25%). After imiquimod treatment, HPV was found in 4 cases (30.7%) and HPV type 16 was found in 2 cases (50%) (Table 7).

The total time to recurrence of HSIL was 19.7 months (range 3.2–32.7). The times to recurrent HSIL in complete, partial and non-response cases are shown in Table 3. 

The time to recurrence in HPV-negative cases post-imiquimod was 19.6 months (1 case), while in HPV-positive cases it was 17.1 months (3.2–32.7).

## 4. Discussion

### 4.1. Response to Imiquimod

A complete response occurred in 50% of vulvar HSIL cases treated with imiquimod, in line with the observations from previous studies. The RCTs by Mathiesen [14] and Trutnovsky [18] reported similar complete response rates at 81% and 80% respectively. Elsewhere, a prospective study reported a complete response of 31% [19] while an RCT reported a rate of 35% [10]. By contrast, a retrospective study reported a much higher complete response rate of 76% [12]. The results of several systematic reviews support this wide variation, reporting a complete response rate from 5% to 100% [8,9,20], with one other reporting a rate of 46% [21].

The discrepancies in response rate likely reflect the different inclusion criteria. For example, the inclusion of vulvar HSIL/VIN 1 can increase the number of responses because vulvar LSIL is not considered a premalignant lesion [7]. Another issue may be the scarcity of patients and follow-up in these studies. We carefully selected cases diagnosed with vulvar HSIL and excluded vulvar LSIL or differentiated VIN to ensure a homogenous sample. Vulvar LSIL is not a premalignant lesion, does not progress to invasive cancer [7], and is associated with low-risk HPV types 6 and 11 in 90% of cases [4]. No studies have evaluated the response of differentiated VIN to imiquimod [4], although differentiated VIN has an HPV-independent development pathway that is less well understood, with inflammation considered a driver of progression to cancer [22]. The differences in response rates to imiquimod could also be explained by differing treatment regimens [18].

Recurrences were frequent (66.6%) in cases with a partial response after an HPV-positive post-treatment biopsy. All the partial responses associated with vulvar HSIL in surgical specimens recurred. Similarly, all 5 patients with HIV infection or immunosuppression showed partial responses and HPV positivity after imiquimod (57% of recurrences occurred in these patients). This is relevant because immunosuppression is a well-known risk factor for HPV persistence in the cervix [23,24] and the development of HPV-related premalignant and invasive cancers [7]. It is also a risk factor for the development of vulvar HSIL [25]. HPV and HIV have close immune interactions, and HIV facilitates HPV infection, possibly due to the impaired clearance of HPV or the reactivation of latent virus [23]. Women with HIV have higher recurrence and progression rates and shorter times to recurrence [26]. We also know that the immunosuppressive drugs used in renal transplantation can increase the carcinogenic effect of HPV [27,28]. One study found a 41-fold increased risk of vulvar cancer compared to the general population [29], while a systematic review reported a higher risk of vulvar cancer in transplanted women compared with the general population [30]. The characteristics of our study, which was retrospective with a small sample size, do not permit us to reach definitive conclusions; however, based on our observations, we suggest the need for a stricter follow-up regime in immunosuppressed patients.

Post-treatment, vulvar HPV was found in 20% of the complete response cases, with only 1 case with HPV 16 type observed. In the partial and non-response cases HPV was positive in 5/6 (83.3%) and in 3/4 (75%) cases respectively. Regarding recurrence post-treatment, HPV was positive in 85.7% of cases that recurred, while it was positive in 30.7% of non-recurrent cases. HPV 16 was positive in 83.3% and 50% of cases with a partial and no response respectively.

When treating cervical intraepithelial neoplasia (CIN) 2–3, the presence of HPV positivity after treatment is strongly associated with CIN persistence or recurrence [31,32]. Indeed, in our study HPV-negative cases were more likely to show complete responses than HPV-positive cases. In a short-term RCT, van Seters demonstrated a strong association between HPV clearance and histological regression of VIN after imiquimod treatment [10]. A retrospective study also reported that the presence of HPV in adjacent tissue after surgery was a risk factor for recurrence [33].

The percentage of HPV type 16 positivity (80%) in this study is consistent with existing data. Vulvar HSIL is produced by high-risk HPV types, specifically related to type 16, in more than 70% of cases [3,34]. We also found HPV 16 in most of the partial responders to imiquimod treatment, non-responders, and recurrences. These results suggest that cases that have an HPV-positive biopsy result after imiquimod treatment should have stricter follow-up after treatment. The infection produced by HPV 16 is associated with a risk of premalignant lesions one order of magnitude higher than other carcinogenic HPV types [35]. 

In our study, 7 patients (35%) experienced recurrences (any type of response), consistent with the literature for treatment with imiquimod or other modalities. In general, the recurrence rates of vulvar HSIL vary between 6% and 50% regardless of the treatment used [5,6,7,36]. After a complete response to imiquimod, recurrence rates in studies varied between 11% in an RCT [10] and 37% in a literature review [8]. However, a systematic review by de Witte indicated a recurrence rate of 33% (range, 0%–100%) [9], while others reported recurrences in 0–37% [8,20].

Factors associated with recurrence in the current study were immunosuppression (*p* = 0.03), previous vulvar HSIL lesions (*p* = 0.01), prior vulvar HSIL surgical treatment (*p* = 0.02), post-imiquimod HPV positivity (*p* = 0.05), and HPV type 16 (*p* = 0.01). It must be taken into account the fact that some patients received additional treatments, and that our study is a case series with a small sample size. Our observations do not allow us to reach a definitive conclusion regarding the effectiveness of imiquimod, but reflect real clinical practice. These observations can be viewed as preliminary results that need to be confirmed in an RCT with an appropriate sample size. 

Although smoking is an established risk factor for developing vulvar HSIL [6,34], we did not find this to be the case, probably because of the small sample size. Our results also showed no association between recurrence or response and age or older age (i.e., 60 years), contrasting with some studies [33] and supporting others [6].

It is important to follow up patients with vulvar HSIL for a long time because of the approximate 10% risk of progression to malignancy [1,7,36]. This risk remains even after surgical treatment, though it falls to 2–4% [36]. Few studies have analyzed the long-term response of vulvar HSIL to imiquimod, which is an important knowledge gap because of the 60% risk of recurrence [1] and 25% risk of late recurrence [33].

Terlou followed patients from the RCT by van Seters for 5 years (median 7.2 years) after treatment with imiquimod and after achieving a complete response at 20 weeks [10,13]. This RCT included both vulvar LSIL and differentiated VIN and found that 8 of the 9 patients with a complete response remained disease-free. In our study, 9 of the 10 cases with a complete response were free of disease at 37 months, with 75% of cases followed for 64.2 months. The case that recurred was also disease-free and HPV-negative after a second treatment.

Despite being recommended as a first-line treatment alongside surgery [4,5,37], the lack of a complete response and frequent recurrences in patients with immune disorders could mean that we should advise against using imiquimod in this group [5]. The antiviral drug cidofovir has achieved an 87% complete response compared to the 78% achieved with imiquimod. Moreover, in an 18-month follow-up period, it was associated with recurrence in 6% of cases compared with 28.4% for imiquimod [38]. Although that study included healthy women, it could still be a viable treatment option in immunosuppressed patients.

Because imiquimod acts through the innate immune system, we do not anticipate a durable protective memory to prevent long-term recurrence or new infection. The topical application also makes action at other anatomic locations unlikely. Thus, durable immunological treatments are needed for vulvar HSIL [39]. Therapeutic vaccines against HPV 16 E6 and E7 oncogenes have been studied, with a phase II study showing a 47% complete response rate [40] and the initial results of an ongoing clinical trial showing a 60% reduction in lesion size [41].

### 4.2. Strengths and Limitations

A key strength of the current study is the restrictive inclusion criteria: only confirmed vulvar HSIL (VIN 2/3) cases treated with imiquimod, excluding vulvar LSIL or differentiated VIN; ensuring a long-term follow-up at the same hospital; and determining vulval HPV status before and after treatment with imiquimod. To our knowledge, no authors have analyzed vulvar HPV in this way for all cases. Although the study is limited by the retrospective design and loss of some data, including missing data, this reflects clinical practice. The small sample size also represents a limitation, but it is difficult to obtain a larger sample because of the low prevalence of vulvar HSIL Finally, the additional treatments administered after imiquimod may have modified the disease outcome. Consequently, the magnitude of effect of the factors associated with response to imiquimod is uncertain.

## 5. Conclusions

Imiquimod for the treatment of vulvar HSIL produced a high response rate (50%) and frequent but generally mild adverse effects after a median follow-up of 37.3 months. Despite an overall recurrence rate of 35%, with higher recurrence rates after a partial response, recurrence was infrequent after a complete response. Ten patients underwent additional treatments following a partial response, no response or recurrences.

All the HIV and immunosuppressed patients treated with imiquimod showed a partial response and required additional treatments. All these patients were HPV-positive post- treatment (100%).

Vulvar HPV was positive in most cases before treatment and remained positive in half of the vulvar HSIL cases after treatment, with HPV type 16 being the most common at both points. Conversely, patients who were HPV-negat + ive post-treatment were more likely to show a complete response.

Recurrence occurred in 35% of cases, with a median time to recurrence of 19.7 months. In those with a complete response, 10% of cases recurred; in those with a partial response, 66.6% recurred, and in those with no response 50% recurred. A total of 57% of these cases were HIV-positive or were immunosuppressed.

A total of 85% of recurrent cases were HPV-positive post-treatment (all were HPV type 16), while 30.7% of non-recurrent cases were HPV-positive post-treatment.

We propose that patients who are immunosuppressed or have an HPV-positive post-treatment result should have a stricter follow-up regime after treatment. Our observations should be confirmed in an RCT with an appropriate sample size.

## Figures and Tables

**Table 1:** Follow-up time and patient characteristics. Patients treated with imiquimod for vulvar high-grade squamous intraepithelial lesion (vulvar HSIL).

	Number of Patients *n* = 20 *n* (%) *	No response *n* = 4 *n* (%) **	Partial Response *n* = 6 *n* (%) **	Complete Response *n* = 10 *n* (%) **	*p*-Value ***
**Patient Characteristics**					
Follow-Up Time (Months) ****					0.944 *****
Median (Min–Max)	37.3 (1–89.1)	42 (1–64.4)	38.2 (17.1–75.9)	37.3 (6.2–89.1)	
IQR	24.3–64.2	16.1–58.6	26.8–69.7	21.9–64	
**Age (Years)**					0.294 *****
Median (Min–Max)	50 (27–87)	53.5 (52–81)	41 (27–70)	47 (28–87)	
**Age (Categorized at 60 Years)**					1.000
<60	16 (80.0)	3 (18.7)	5 (31.3)	8 (50.0)	
≥60	4 (20.0)	1 (25.0)	1 (25.0)	2 (50.0)	
**Smokers**					0.687
No	7 (35.0)	1 (14.3)	2 (28.6)	4 (57.1)	
Yes	11 (55.0)	3 (27.2)	4 (36.4)	4 (36.4)	
Unknown	2 (10.0)	0 (0.0)	0 (0.0)	2 (100.0)	
**HIV Status**					0.111
No	18 (90.0)	4 (22.2)	4 (22.2)	10 (55.6)	
Yes	2 (10.0)	0 (0.0)	2 (100.0)	0 (0.0)	
**Immunosuppression**					0.021
No	17 (85.0)	4 (23.5)	3 (17.6)	10 (58.8)	
Yes	3 (15.0)	0 (0.0)	3 (100.0)	0 (0.0)	
**Previous Vulvar HSIL Lesion**					0.086
No	11 (55.0)	1 (9.1)	2 (18.2)	8 (72.7)	
Yes	9 (45.0)	3 (33.3)	4 (44.5)	2 (22.2)	
**Previous Vulvar HSIL Surgery**					0.060
No	13 (65.0)	2 (15.4)	2 (15.4)	9 (69.2)	
Yes	7 (35.0)	2 (28.6)	4 (57.1)	1 (14.3)	
**Total**	20 (100.0)	4 (20.0)	6 (30.0)	10 (50.0)	

IQR, interquartile range (25–75%); HIV, human immunodeficiency virus; vulvar HSIL, vulvar high-grade squamous intraepithelial lesion. * Column percentage. ** Row percentage. *** Fisher exact test *p*-value. **** 10 cases received additional treatment, recurrent vulvar HSIL was observed in 7 cases, and 3 cases were diagnosed as squamous carcinoma of the vulva. ***** Kruskal-Wallis test *p*-value.

**Table 2:** Lesion characteristics pre-post imiquimod treatment. Patients treated with imiquimod for vulvar high-grade squamous intraepithelial lesion (vulvar HSIL).

	Number of Patients *n* = 20 *n* (%) *^1^	No Response *n* = 4 *n* (%) **	Partial Response *n* = 6 *n* (%) **	Complete Response *n* = 10 *n* (%) **	*p*-Value ***
**Lesion Characteristics Pre Imiquimod**					
**HPV Status**					1.000
Negative	1 (5.0)	0 (0.0)	0 (0.0)	1 (100.0)	
Positive	19 (95.0)	4 (21.0)	6 (31.6)	9 (47.4)	
**HPV Type**					0.777
Negative	1 (5.0)	0 (0.0)	0 (0.0)	1 (100.0)	
Type 16	16 (80.0)	3 (18.7)	6 (37.5)	7 (43.8)	
Other HR-HPV types	3 (15.0)	1 (33.3)	0 (0.0)	2 (66.7)	
**Type of Lesion**					0.897
Unifocal	5 (25.0)	1 (20.0)	2 (40.0)	2 (40.0)	
Isolated multifocal	6 (30.0)	2 (33.3)	1 (16.7)	3 (50.0)	
Multifocal	9 (45.0)	1 (11.1)	3 (33.3)	5 (55.6)	
**Maximum Diameter of Lesion (mm)**					0.143 ****
Median (Min-Max)	20 (5–60)	11.5 (7–20)	22 (15–30)	21 (5–60)	
**Lesion Characteristics Post-Imiquimod**					
**HPV Status**					**0.030**
Negative	10 (50.0)	1 (10.0)	1 (10.0)	8 (80.0)	
Positive	10 (50.0)	3 (30.0)	5 (50.0)	2 (20.0)	
**HPV Type**					**0.012**
Negative	10 (50.0)	1 (10.0)	1 (10.0)	8 (80.0)	
Type 16	8 (40.0)	2 (25.0)	5 (62.5)	1 (12.5)	
Other HR-HPV types	2 (10.0)	1 (50.0)	0 (0.0)	1 (50.0)	
**Histology Result**					**0.000**
Vulvar epithelium without pathology	9 (45.0)	0 (0.0)	0 (0.0)	9 (100.0)	
Vulvar LSIL	1 (5.0)	0 (0.0)	0 (0.0)	1 (100.0)	
Vulvar HSIL	9 (45.0)	3 (33.3)	6 (66.7)	0 (0.0)	
Squamous carcinoma of the vulva	1 (5.0)	1 (100.0)	0 (0.0)	0 (0.0)	
**Type of Lesion**					0.329
No lesion	3 (15.0)	0 (0.0)	0 (0.0)	3 (100.0)	
Unifocal	7 (35.0)	1 (14.3)	2 (28.6)	4 (57.1)	
Isolated multifocal	7 (35.0)	1 (14.3)	3 (42.9)	3 (42.9)	
Multifocal	3 (15.0)	2 (66.7)	1 (33.3)	0 (0.0)	
**Maximum Diameter of Lesion (mm)**					0.015 ****
Median (Min-Max)	10 (0–80)	10 (10–80)	8.5 (3–20)	0 (0–0)	
Recurrent Vulvar HSIL *****					0.060
No	13 (65.0)	2 (15.4)	2 (15.4)	9 (69.2)	
Yes	7 (35.0)	2 (28.6)	4 (57.1)	1 (14.3)	
**Total**	20 (100.0)	4 (20.0)	6 (30.0)	10 (50.0)	

HPV, human papilloma virus; HR-HPV, high-risk human papilloma virus; vulvar HSIL, vulvar high-grade squamous intraepithelial lesion; vulvar LSIL, vulvar low-grade squamous intraepithelial lesion. Column percentage. **^1^** In total, 10 cases received additional treatment. * Column percentage. ** Row percentage. *** Fisher exact test *p*-value. **** Kruskal-Wallis test *p*-value. ***** In 1/10 of cases with complete response, 4/6 of cases with partial response, and 2/4 of cases with no response to imiquimod.

**Table 3:** Follow up time, biopsy time post imiquimod, and time of diagnosis of recurrent vulvar HSIL in patients with complete, partial and no response to treatment with imiquimod.

Type of Response: *n* (%)	Follow Up Time (Months) *	Biopsy Time (Months) **	Time of Diagnosis of Recurrent Vulvar HSIL (Months) ***	Duration of Treatment (Weeks)
	Median (Min–Max)	Median (Min–Max)	Median (Min–Max)	Median (Min–Max)
Complete response: 10 (50.0)	37.3 (6.2–89.1)	4.7 (1.0–20.9)	19.7 ****	13.0 (4.0–20)
Partial response: 6 (30.0)	38.2 (17.1–75.9)	5.8 (3.6–37.5)	16.2 (3.2–32.7)	16.5 (15.0–32.0)
No response: 4 (20.0)	42.0 (1.0–64.4)	3.3 (1.1–11.1)	22.1 (12.0–32.2) *****	9.0 (4.0–15.0)
**Total:** 20 (100.0)	37.3 (1.0–89.1)	4.9 (1.0–37.5)	19.7 (3.2–32.7)	14.5 (4.0–32.0)

* 10 cases received additional treatment, recurrent vulvar HSIL was observed in 7 cases, and 3 cases were diagnosed as squamous carcinoma of the vulva. ** Response time to imiquimod. *** Recurrent vulvar HSIL occurred in 1/10 of cases with complete response, 4/6 of cases with partial response, and 2/4 of cases with no response to imiquimod. **** In 1 case. ***** In 2 cases.

**Table 4:** Imiquimod side effects and withdrawal. Patients treated with imiquimod for vulvar high-grade squamous intraepithelial lesion (vulvar HSIL).

	Number of Patients *n* = 20 *n* (%) *	No Response *n* = 4 *n* (%) **	Partial Response *n* = 6 *n* (%) **	Complete Response *n* = 10 *n* (%) **	*p*-Value ***
**Imiquimod Side effects and Withdrawal**					
**Imiquimod Side Effect_Signs**					0.940
No	8 (40.0)	1 (12.5)	3 (37.5)	4 (50.0)	
Erythema	6 (30.0)	2 (33.3)	2 (33.3)	2 (33.3)	
Erosion	2 (10.0)	0 (0.0)	1 (50.0)	1 (50.0)	
Depigmentation	1 (5.0)	0 (0.0)	0 (0.0)	1 (100.0)	
Other	3 (15.0)	1 (33.3)	0 (0.0)	2 (66.7)	
**Imiquimod Side Effect_Symptoms**					0.227
No	9 (45.0)	2 (22.2)	2 (22.2)	5 (55.6)	
Pain	2 (10.0)	1 (50.0)	1 (50.0)	0 (0.0)	
Pain, stinging	2 (10.0)	1 (50.0)	0 (0.0)	1 (50.0)	
Burning	2 (10.0)	0 (0.0)	2 (100.0)	0 (0.0)	
Other	5 (25.0)	0 (0.0)	1 (20.0)	4 (80.0)	
**Reduction of Doses**					1.000
No	18 (90.0)	4 (22.2)	5 (27.8)	9 (50.0)	
Yes	2 (10.0)	0 (0.0)	1 (50.0)	1 (50.0)	
**Cessation of Treatment**					0.629
No	5 (25.0)	1 (20.0)	3 (60.0)	1 (20.0)	
Yes, temporary	10 (50.0)	2 (20.0)	2 (20.0)	6 (60.0)	
Yes, definitely	5 (25.0)	1 (20.0)	1 (20.0)	3 (60.0)	
**Total**	20 (100.0)	4 (20.0)	6 (30.0)	10 (50.0)	

* Column percentage. ** Row percentage. *** Fisher exact test *p*-value.

**Table 5:** Patient and lesion characteristics in cases with and without additional treatment after treatment with imiquimod.

		Additional Treatment *	Number of Patients *n* = 20 *n* (%) **	No Response *n* = 4 *n* (%) ***	Partial Response *n* = 6 *n* (%) ***	Complete Response *n* = 10 *n* (%) ***	*p*-Value ****
**Patient Characteristics**							
Immunosuppression	No	No	10 (50.0)	1 (10.0)	-	9 (90.0)	**0.004**
		Yes	7 (35.0)	3 (42.9)	3 (42.9)	1 (14.3)	
	Yes	No	-	-	-	-	-
		Yes	3 (15.0)	-	3 (100.0)	-	
Previous vulvar HSIL lesion	No	No	9 (45.0)	1 (11.1)	-	8 (88.9)	**0.018**
		Yes	2 (10.0)	-	2 (100.0)	-	
	Yes	No	1 (5.0)	-	-	1 (100.0)	0.222
		Yes	8 (40.0)	3 (37.5)	4 (50.0)	1 (12.5)	
Previous vulvar HSIL surgery	No	No	9 (45.0)	1 (11.1)	-	8 (88.9)	**0.052**
		Yes	4 (20.0)	1 (25.0)	2 (50.0)	1 (25.0)	
	Yes	No	1 (5.0)	-	-	1 (100.0)	0.143
		Yes	6 (30.0)	2 (33.3)	4 (66.7)	-	
**Lesion Characteristics Post Imiquimod**							
HPV status	Negative	No	8 (40.0)	1 (12.5)	-	7 (87.5)	0.378
		Yes	2 (10.0)	-	1 (50.0)	1 (50.0)	
	Positive	No	2 (10.0)	-	-	2 (100.0)	**0.022**
		Yes	8 (40.0)	3 (37.5)	5 (62.5)	-	
HPV types	Negative	No	8 (40.0)	1 (12.5)	-	7 (87.5)	0.378
		Yes	2 (10.0)	-	1 (50.0)	1 (50.0)	
	Type 16	No	1 (5.0)	-	-	1 (100.0)	0.125
		Yes	7 (35.0)	2 (28.6)	5 (71.4)	-	
	Other HR-HPV	No	1 (50.0)	-	-	1 (100.0)	1.000
		Yes	1 (50.0)	1 (100.0)	-	-	
Recurrent vulvar HSIL *****	No	No	10 (50.0)	1 (10.0)	-	10 (90.0)	**0.014**
		Yes	3 (15.0)	1 (33.3)	2 (66.7)	-	
	Yes	No	-	-	-	-	-
		Yes	7 (35.0)	2 (28.6)	4 (57.1)	1 (14.3)	

Vulvar HSIL, vulvar high-grade squamous intraepithelial lesion; HPV, human papilloma virus. * 10 cases received additional treatment. ** Column percentage. *** Row percentage. **** Fisher exact test *p*-value. ***** In 1/10 of cases with complete response, 4/6 of cases with partial response, and 2/4 of cases with no response to imiquimod.

**Table 6:** Recurrent vulvar HSIL in cases with and without additional treatment after treatment with imiquimod.

		Additional Treatment *	Number of Patients *n* = 20 *n* (%) **	No Recurrent Vulvar HSIL *n* = 13 *n* (%) ***	Recurrent Vulvar HSIL *n* = 7 *n* (%) ***^1^	*p*-Value ****
**Patient Characteristics**						
Immunosuppression	No	No	10 (50.0)	10 (100.0)	-	**0.015**
		Yes	7 (35.0)	3 (42.9)	4 (57.1)	
	Yes	No	-	-	-	-
		Yes	3 (15.0)	-	3 (100.0)	
Previous vulvar HSIL lesion	No	No	9 (45.0)	9 (100.0)	-	0.182
		Yes	2 (10.0)	1 (50.0)	1 (50.0)	
	Yes	No	1 (5.0)	1 (100.0)	-	0.333
		Yes	8 (40.0)	2 (25.0)	6 (75.0)	
Previous vulvar HSIL surgery	No	No	9 (45.0)	9 (100.0)	-	0.077
		Yes	4 (20.0)	2 (50.0)	2 (50.0)	
	Yes	No	1 (5.0)	1 (100.0)	-	0.286
		Yes	6 (30.0)	1 (16.7)	5 (83.3)	
**Lesion Characteristics Post Imiquimod**						
HPV status	Negative	No	8 (40.0)	8 (100.0)	-	0.200
		Yes	2 (10.0)	1 (50.0)	1 (50.0)	
	Positive	No	2 (10.0)	2 (100.0)	-	0.133
		Yes	8 (40.0)	2 (25.0)	6 (75.0)	
HPV type	Negative	No	8 (40.0)	8 (100.0)	-	0.200
		Yes	2 (10.0)	1 (50.0)	1 (50.0)	
	Type 16	No	1 (5.0)	1 (100.0)	-	0.250
		Yes	7 (35.0)	1 (14.3)	6 (85.7)	
	Other HR-HPV	No	1 (5.0)	1 (100.0)	-	-
		Yes	1 (5.0)	1 (100.0)	-	

Vulvar HSIL, vulvar high-grade squamous intraepithelial lesion; HPV, human papilloma virus. * 10 cases received additional treatment. ** Column percentage. *** Row percentage. **** Fisher exact test *p*-value. **^1^** In 1/10 of cases with complete response, 4/6 of cases with partial response, and 2/4 of cases with no response to imiquimod.

**Table 7:** HPV status and type of HR-HPV in the biopsy pre- and post- imiquimod in patients with or without recurrent vulvar HSIL.

			Recurrent Vulvar HSIL *n* (%)		
			No *n* = 13 (65.0)	Yes *n* = 7 (35.0) *	Total *n* = 20 (100.0) **
**HPV Pre-Imiquimod**	**HPV status**	**Negative**	1 (7.7)	-	1 (5.0)
		**Positive**	12 (92.3)	7 (100.0)	19 (95.0)
	**Type of HR-HPV**	**Type 16**	9 (75.0)	7 (100.0)	16 (84.2)
		**Other HR-HPV**	3 (25.0)	-	3 (15.7)
**HPV Post-Imiquimod**	**HPV status**	**Negative**	9 (69.2)	1 (14.3)	10 (50.0)
		**Positive**	4 (30.7)	6 (85.7)	10 (50.0)
	**Type of HR-HPV**	**Type 16**	2 (50.0)	6 (100.0)	8 (80.0)
		**Other HR-HPV**	2 (50.0)	-	2 (20.0)

HPV, human papilloma virus; HR-HPV, high-risk human papilloma virus. * In 1/10 of cases with complete response, 4/6 of cases with partial response, and 2/4 of cases with no response to imiquimod. ** 10 cases received additional treatment.

**Table 8:** Recurrent vulvar high-grade squamous intraepithelial lesion (vulvar HSIL) after treatment with imiquimod in patients with vulvar HSIL.

	Number of Patients *n* = 20 *n* (%) *^1^	No Recurrent Vulvar HSIL *n* = 13 *n* (%) **	Recurrent Vulvar HSIL *n* = 7 *n* (%) **^2^	*p*-Value ***
**Patient Characteristics**				
**Immunosuppression**				**0.031**
No	17 (85.0)	13 (76.5)	4 (23.5)	
Yes	3 (15.0)	0 (0.0)	3 (100.0)	
**Previous Vulvar HSIL Lesion**				**0.017**
No	11 (55.0)	10 (90.9)	1 (9.1)	
Yes	9 (45.0)	3 (33.3)	6 (66.7)	
**Previous Vulvar HSIL Surgery**				**0.022**
No	13 (65.0)	11 (84.6)	2 (15.4)	
Yes	7 (35.0)	2 (28.6)	5 (71.4)	
**Lesion Characteristics Post Imiquimod**				
**HPV Status**				0.057 ****
Negative	10 (50.0)	9 (90.0)	1 (10.0)	
Positive	10 (50.0)	4 (40.0)	6 (60.0)	
**HPV Type**				**0.010**
Negative	10 (50.0)	9 (90.0)	1 (10.0)	
Type 16	8 (40.0)	2 (25.0)	6 (75.0)	
Other HR-HPV types	2 (10.0)	2 (100.0)	0 (0.0)	
**Histology Result**				**0.028**
Vulvar epithelium without pathology	9 (45.0)	8 (88.9)	1 (11.1)	
Vulvar LSIL	1 (5.0)	1 (100.0)	0 (0.0)	
Vulvar HSIL	9 (45.0)	3 (33.3)	6 (66.7)	
Squamous carcinoma of the vulva	1 (5.0)	1 (100.0)	0 (0.0)	
**Total**	20 (100.0)	13 (65.0)	7 (35.0)	

Vulvar HSIL, vulvar high-grade squamous intraepithelial lesion; vulvar LSIL, vulvar low-grade squamous intraepithelial lesion; HPV, human papilloma virus; HR-HPV, high-risk human papilloma virus. * Column percentage. ** Row percentage. *** Fisher exact test *p*-value. **^1^** 10 cases received additional treatment. **^2^** In 1/10 of cases with complete response, 4/6 of cases with partial response, and 2/4 of cases with no response to imiquimod.

## Data Availability

The data presented in this study are available on request from the corresponding author. The data are not publicly available due to the localization of the database in the intranet of Bellvitge Hospital.

## Abbreviations

ECSVD	European College for the Study of Vulval Disease
EFC	European Federation for Colposcopy
ESGO	European Society of Gynaecological Oncology
FFPE	Formalin fixed paraffin Embedded
HSIL	High-grade squamous intraepithelial lesion
ISSVD	International Society for the Study of Vulvovaginal Disease
VIN	Vulvar intraepithelial neoplasia
